# Effect of trimetazidine on left ventricular functions and cardiac biomarkers in diabetic patients with left ventricular diastolic dysfunction: a randomized controlled trial

**DOI:** 10.1038/s41598-024-83213-w

**Published:** 2025-01-16

**Authors:** Heba Serag, Lamia El Wakeel, Viola William, Mona Abdelsalam, Ahmed Abdelsalam, Rana Sayed

**Affiliations:** 1 Department of Clinical Pharmacy, Faculty of Pharmacy, Ain Shams University, Cairo, Egypt; 2 Department of Cardiology, Faculty of Medicine, Ain Shams University, Cairo, Egypt; 3 Department of Internal medicine, Endocrinology and Metabolism, Faculty of Medicine, Ain Shams University, Cairo, Egypt

**Keywords:** Trimetazidine, Diabetic cardiomyopathy, T2DM, Diastolic dysfunction, GLS, NT-proBNP, Cardiology, Diseases, Endocrinology, Medical research

## Abstract

We investigated the impact of trimetazidine treatment on left ventricular (LV) functions and cardiac biomarkers in diabetic patients with diastolic dysfunction as an early stage of diabetic cardiomyopathy. Sixty-three patients were randomly assigned to receive either trimetazidine or a placebo for 3 months. At baseline and after 3-months of treatment, measurements of serum levels of glycemic control parameters, lipid profile, tumor necrosis factor alpha, transforming growth factor beta 1, n-terminal pro brain natriuretic peptide and assessment of modified Medical Research Council (mMRC) dyspnea score, echocardiographic indices of LV functions and LV global longitudinal strain (GLS) were performed. After 3-months, trimetazidine group exhibited a significant reduction in left atrial volume index by 6.99% versus an increase by 0.66% in placebo group, p = 0.034. Moreover, average e’ increased by a significantly higher percentage in trimetazidine versus placebo group (8.46 ± 18.64 vs. -2.49 ± 14.52, respectively. *p* = 0.015). Trimetazidine treatment resulted in a significant increase in LVGLS by 6.66% versus LVGLS reduction by 2.79% in placebo group (*p* = 0.004). Trimetazidine group had a significantly lower median mMRC dyspnea score compared to placebo (0 vs. 1, respectively, *p* = 0.015) and a significantly lower low-density lipoprotein (LDL-C) (103.43 ± 28.31 vs. 125.34 ± 45.27, respectively, *p* = 0.032). There was no significant difference between both groups in levels of other biomarkers. Three-months treatment with trimetazidine improved diastolic function parameters, LVGLS, dyspnea severity and LDL-C levels in diabetic patients with diastolic dysfunction.

**Supplementary Information:**

The online version contains supplementary material available at 10.1038/s41598-024-83213-w.

## Introduction

Diabetic cardiomyopathy (DCM) is defined as cardiomyopathy in patients with diabetes mellitus (DM) in the absence of coronary artery disease, valvular disease, and hypertension^1^. The prevalence of DCM ranges from 11.7 to 67.0% depending on the diagnostic criteria^2^. DCM progresses through a silent, asymptomatic subclinical phase that is typically prolonged and characterized by cellular structural damage that results in diastolic dysfunction, systolic dysfunction, and finally heart failure^3^. The prevalence of left ventricular (LV) diastolic dysfunction among diabetic patients was found to be 23 − 75%^4–7^. The probability of heart failure development at 5 years for diabetic patients with diastolic dysfunction was 36.9% and posed a 2.5 times higher mortality risk compared to those without LV diastolic dysfunction^6^. Early diagnosis of DCM is still difficult because no clear definition for the disease is available, thus no clear diagnostic criteria have been described^8^.

The pathophysiology of DCM is complex and involves multiple metabolic pathways, including altered fatty acid metabolism^9^, oxidative stress, persistent neurohormonal activation^10^, inflammation, and increased transforming growth factor- β (TGF-β) activity brought on by chronic hyperglycemia^11^. In healthy hearts, fatty acids (FAs) are the preferred fuel substrates and are responsible for producing about 70–90% of myocardial adenosine triphosphate (ATP). While in type 2 DM, despite systemic hyperglycemia, the myocardial preference for FAs as energetic substrates increases. Unfortunately, FAs oxidation requires more oxygen to produce the same amount of ATP compared to glucose oxidation, thus, increased FA oxidation reduces cardiac efficiency (calculated as cardiac work divided by myocardial oxygen consumption)^9^.

Trimetazidine (TMZ) is a well-tolerated drug primarily used to treat angina pectoris^12^. It acts by inhibiting the mitochondrial long-chain 3-ketoacyl-CoA thiolytic enzyme; a key enzyme that catalyzes the last step of the FA β-oxidation cycle, that results in reduction of the rate of FA aerobic oxidation and indirectly increases the activity of pyruvate dehydrogenase, which is the rate-limiting enzyme of glucose aerobic oxidation^13^. Moreover, TMZ may potentially protect against DCM through its antioxidant properties^14^, anti-inflammatory effect^15^, and ability to alleviate myocardial fibrosis^16,17^. Beyond the substantial positive data regarding the metabolic and clinical benefits of TMZ, more importantly, it has no significant hemodynamic effect and mild tolerable side effects^12^. Hence, TMZ may play a role in the treatment of early DCM.

We hypothesized that TMZ may have a potential effect on left ventricular functions and cardiac inflammatory and fibrosis biomarkers of diabetic patients with LV diastolic dysfunction as an early stage of DCM.

## Methods

### Study design

This study was a prospective, randomized, single blinded, placebo-controlled study that was registered in clinicaltrials.gov on 27/09/2022 (registration number: NCT05556005). We used the CONSORT reporting guidelines in writing this manuscript^18^.

#### Patients’ recruitment

The study population consisted of patients with type 2 DM. Patients were recruited from the out-patient clinic of the Department of Internal Medicine - Endocrinology unit, Ain Shams University, Cairo, Egypt. Patients presenting to the clinic were assessed for eligibility according to the following inclusion and exclusion criteria:

*Inclusion criteria* included DM outpatients aged 40–75 years with left ventricular ejection fraction (EF) ≥ 50% and echocardiographic evidence of LV diastolic dysfunction.

LV diastolic dysfunction was defined as reduced lateral or septal peak early diastolic velocity of mitral annular motion (e’) for age or left atrial volume index (LAVI) > 28 ml/m^2^^19^. This definition of LV diastolic dysfunction was chosen to specifically target patients with early subclinical DCM who might be more responsive to TMZ treatment.

*Exclusion criteria* included the presence of valvular, congenital or ischemic heart disease, inadequately controlled hypertension (defined as systolic blood pressure (SBP) > 140 mm Hg or diastolic blood pressure (DBP) > 90 mm Hg), glycated hemoglobin (HbA1c) < 10%, history of intolerance or allergic response to TMZ, severe liver dysfunction or severe renal dysfunction (defined as creatinine clearance < 30 ml/min), other significant comorbidities such as malignancy, significant psychiatric illness, autoimmune diseases, thyroid gland disorders, Parkinson’s disease or motor disorders, pregnancy, and breast feeding.

Upon enrollment, only 63 patients were eligible and were randomly assigned using a 4-sized block randomization to one of the following two groups: TMZ group (*n* = 32); received 35 mg of TMZ modified release tablet twice daily in addition to their standard treatment for three months. Placebo group (*n* = 31); received placebo tablet of matched appearance and taste twice daily in addition to their standard treatment for three months.

#### ***Ethical consideration***

The study protocol was revised and approved by the ethics committee of the Faculty of Pharmacy, Ain Shams University (ACUC-FP-ASU). Patients were educated about the study protocol and an informed consent was obtained from each patient prior to enrollment in the study and the research was conducted in accordance with the Declaration of Helsinki.

##### Baseline evaluation

###### Demographic, anthropometric, and medical history

Demographic, anthropometric and medical history data were collected from all subjects and included age, sex, basic anthropometry to establish body mass index (BMI) and body surface area (BSA), smoking status, duration of diabetes, comorbidities, and medication history.

## Blood sample collection and laboratory assay

Peripheral venous blood sample was drawn after 10–12 h fasting, and biochemical analysis was performed at baseline and at the end of the study by a clinical laboratory specialist blinded to the study groups. HbA1c levels were determined using quantitative turbidimetric method and fasting blood glucose (FBG) levels were assayed using glucose oxidase method. Total cholesterol, triglycerides and high-density lipoprotein (HDL-C) levels were measured by enzymatic colorimetric method and low-density lipoprotein (LDL-C) levels were calculated indirectly using Friedewald Equation: *LDL-C = Total Cholesterol– HDL-C – Triglycerides/5*.

Serum samples were stored at −80 °C for later simultaneous analysis of tumor necrosis factor alpha (TNF-α) and TGF-β1 using Enzyme Linked Immunosorbent Assay (ELISA) technique using commercial kits manufactured by bio-technology laboratory, Zhejiang, China, (Catalogue number E0082HU and E0134Hu, respectively) and N- terminal pro brain natriuretic peptide (NT-proBNP) using a kit manufactured by SunRed biological technology company, Shanghai, China, (Catalogue number 201-12-1240).

## Dyspnea assessment

The degree of dyspnea was assessed by a physician blinded to the study groups using the Modified Medical Research Council (mMRC) dyspnea scale. It is a self-rating scale used to assess the level of limitation that dyspnea causes in day-to-day activities, and it ranges from 0 to 4: 0, no breathlessness except on strenuous exercise and 4, too breathless to leave the house, or breathless when dressing or undressing^20^.

## Complete echocardiographic study

A Complete transthoracic echocardiogram was performed using vivid E95 cardiac ultrasound manufactured by GE healthcare, United States. The images were acquired while the patient was in the left lateral position with good ECG gating. All echocardiographic measurements were performed by a single operator blinded to the study groups. All echocardiographic measurements were obtained according to the standards set out by the American Society of Echocardiography and the European Association of Cardiovascular Imaging from the standard apical and parasternal views^21^. M-mode was used to measure the maximal left atrial internal diameter (LAD), left ventricular end-diastolic dimensions (LVEDD), left ventricular end-systolic dimensions (LVESD), inter- ventricular septal wall thickness (IVS) in diastole and left ventricular posterior wall thickness (PWT) in diastole. Left ventricular mass (LVM) was calculated using Devereux’s formula^22^, then divided by BSA to give LVM index. Using two-dimensional (2D) imaging, LAV was measured just at the end of LV systole from the apical four and two chamber views and LAVI was calculated by indexing LAV to BSA. LV volumes were measured at end-diastole and end-systole, from apical four and two chamber views then LVEF was calculated using modified Simpson’s method. LV volumes were indexed to BSA. The velocity of early (E) and late (A) diastolic LV filling as well as the deceleration time of the E-wave (DT) were measured from the transmitral pulsed Doppler waves and the E/A ratio was subsequently calculated. Tissue Doppler imaging provided velocity of relaxation of the myocardial wall at both the septal and lateral mitral annuli and provided a corresponding early (e’) wave. The average of the septal and lateral e’ waves was used in analyses to calculate the average E/e’. Left ventricular global longitudinal strain (LVGLS) was assessed by 2D speckle tracking echocardiography (STE) as follows, cine loops were stored from the apical window for apical 4-chamber, 3-chamber and 2-chamber views with good ECG gating. Offline analysis was then performed using EchoPAC 202 GE software. A region of interest was traced on the endocardium at end-systole with a point-and-click approach for the apical views. A second larger region of interest near the epicardium was then generated and manually adjusted. Each apical image was divided into six standard segments, and six corresponding time-strain curves were generated. GLS was determined as the averaged peak strain of segments from the apical view.

### Follow-up evaluation

All patients were followed up at regular intervals monthly and were asked to report the occurrence of any undesirable side effects. Compliance to TMZ was assessed using pill counting method. Patients who failed to consume more than 80% of their capsules were considered non-compliant^23^. After three months of treatment, all patients were reassessed for all laboratory, clinical and echocardiographic parameters.

## Statistical methods

Data management and analysis were performed using Statistical Package for Social Sciences (SPSS) V. 20. Numerical data were summarized using means and standard deviations or medians and inter-quartile ranges. Categorical data were summarized as percentages and frequency. Data were explored for normality using Kolmogorov-Smirnov and Shapiro-Wilk tests. Comparisons between the trimetazidine and placebo groups were done using unpaired Student t-test for continuous parametric variables or Mann-Whitney test for non-parametric variables, at each time point. Changes from baseline to 3 months for each group were tested using the paired Student t-test for continuous parametric variables or Wilcoxon signed rank test for non-parametric variables. Chi-square and Fisher’s exact tests were used to compare between the groups with respect to categorical data. Parametric (Pearson r) and non-parametric (Spearman rho) correlation coefficients were used to examine cross-sectional associations between variables. A multiple linear regression analysis was performed to examine the association between post-treatment measured parameters and the type of intervention (TMZ or placebo). Baseline variables that were statistically different between the two study groups or deemed clinically significant were incorporated into the multivariate models as covariates. All p-values were two-sided. p-values < 0.05 were considered significant.

## Sample size calculation

The required sample size was calculated based on data from a previous study on the effect of TMZ on E/A ratio^24^. G power version 3.1 statistical software was used. Assuming an effect size of 1, a sample size of 23 patients per group was required to achieve a power of 0.9 while keeping alpha level at 0.05. Considering a 30% drop-out, the sample size was decided to be 60 subjects. (30 patients for each group)

## Results

**Fig. 1:**
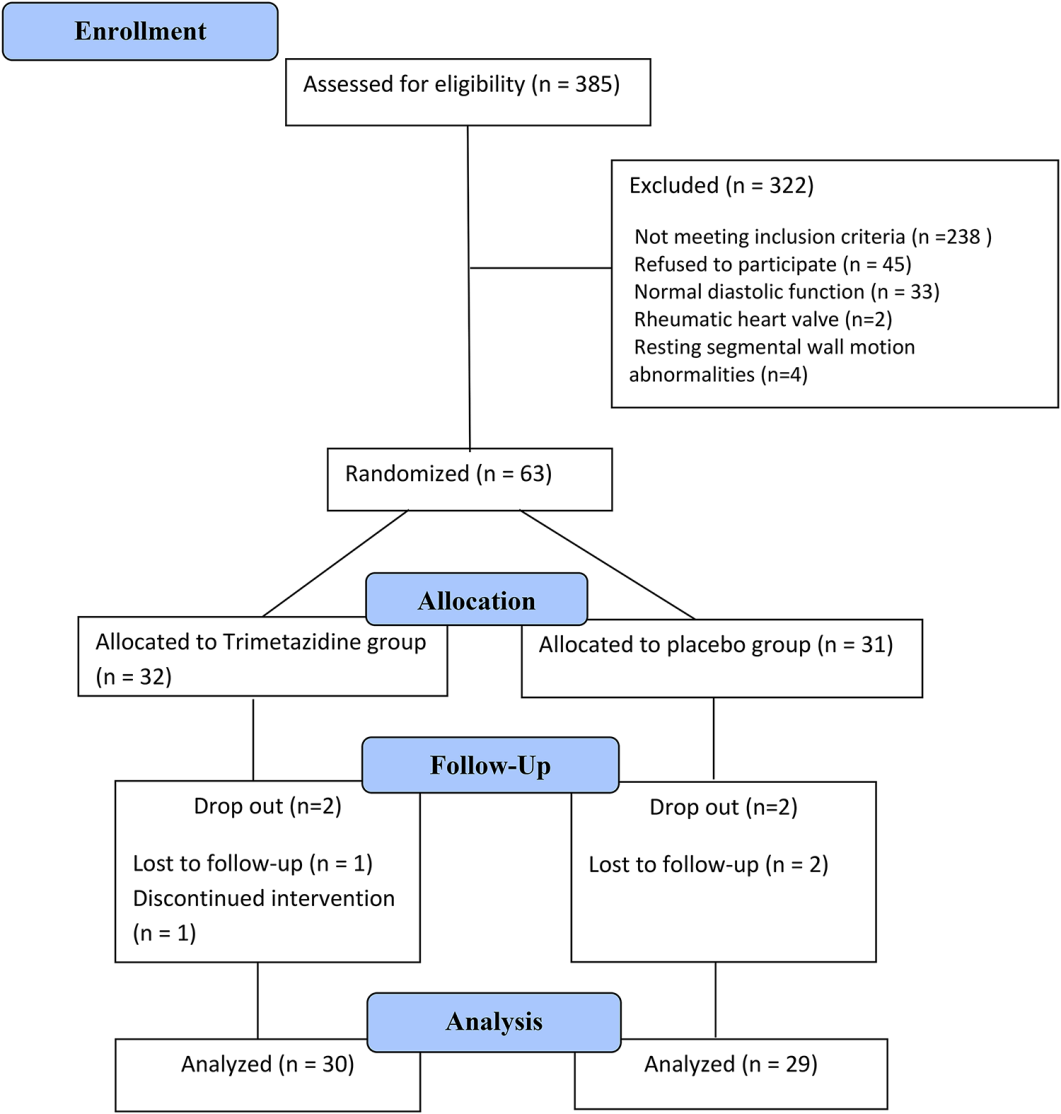
CONSORT diagram representing the enrollment, allocation, follow-up and the analysis process.

From June 2022 till January 2024, three-hundred and eighty-five diabetic patients were assessed for eligibility. Echocardiography was done for 102 patients out of whom 33 patients had normal LV diastolic function, 4 patients had evidence of resting segmental wall motion abnormalities, 2 patients had significant valvular disease and only 63 patients fulfilled the eligibility criteria and were enrolled in the study from which 4 patients were dropped out during the study (2 drop out from trimetazidine group and 2 drop out from placebo group) and only 59 patients continued to the end of the study. (Fig. 1)

### Demographic and clinical characteristics

Baseline demographic and clinical features of both groups were comparable with respect to age, gender distribution, BMI, BSA, smoking status and duration of diabetes. The percentage of patients with hypertension and dyslipidemia were comparable between the two study groups, and no significant differences were observed in the medications used. (Table 1)

**Table 1:** Demographic data and clinical characteristics of the study groups.

Variables	TMZ group (*n* = 30)	Placebo group (*n* = 29)	*p*-value
Age (Years)	58.60 ± 6.589	56.48 ± 7.347	0.248^a^
BMI (kg/m^2^)	32.61 ± 5.21	31.13 ± 4.84	0.26^a^
BSA (m^2^)	1.89 ± 0.14	1.92 ± 0.22	0.54^a^
Gender (M/F), n	6/24	7/22	0.701^b^
Smokers, n (%)	3 (10)	3 (10.3)	1^c^
Duration of diabetes (Years)	12.18 ± 7.25	12.91 ± 7.46	0.707^a^
HTN, n (%)	10 (33.3)	16 (55.2)	0.091^b^
Duration of HTN (Years)	9.65 ± 5.83	7.84 ± 8.31	0.554^a^
Dyslipidemia, n (%)	21(70)	17(58.6)	0.361^a^
Medications			
Insulin, n (%)	20 (66.7)	17 (58.6)	0.523^a^
Metformin, n (%)	18 (60)	22 (75.9)	0.192^a^
Sulphonyl urea, n (%)	10 (33.3)	9 (31)	0.85^a^
DPP4-inhibitors, n (%)	6 (20)	10 (34.5)	0.211^a^
Statin, n (%)	21 (70)	17 (58.6)	0.361^a^
β-blockers, n (%)	4 (13.3)	8 (27.6)	0.174^c^
CCB, n (%)	4 (13.3)	4 (13.8)	1.00^a^
ACE-inhibitors, n (%)	7 (22.3)	6 (20.7)	0.807^c^
ARBs, n (%)	1 (3.3)	6 (20.7)	0.052^c^
Thiazide, n (%)	2 (6.7)	4 (13.8)	0.424^a^
Aspirin, n (%)	12 (40)	10 (34.5)	0.661^a^

Data for continuous variables given as mean ± standard deviation; BMI: body mass index; BSA: body surface area; M/F: male/female; n: number; HTN: hypertension; DPP-4: dipeptidyl peptidase; CCB: calcium channel blocker; ACE: angiotensin converting enzyme; ARBs: angiotensin II receptor blockers.

Statistical test: ^a^ Unpaired student t-test, p-value ≥ 0.05; ^b^ Chi-square test, p-value ≥ 0.05; ^c^ Fisher’s exact test, p-value ≥ 0.05.

### Glycemic control and lipid profile parameters

**Fig. 2:**
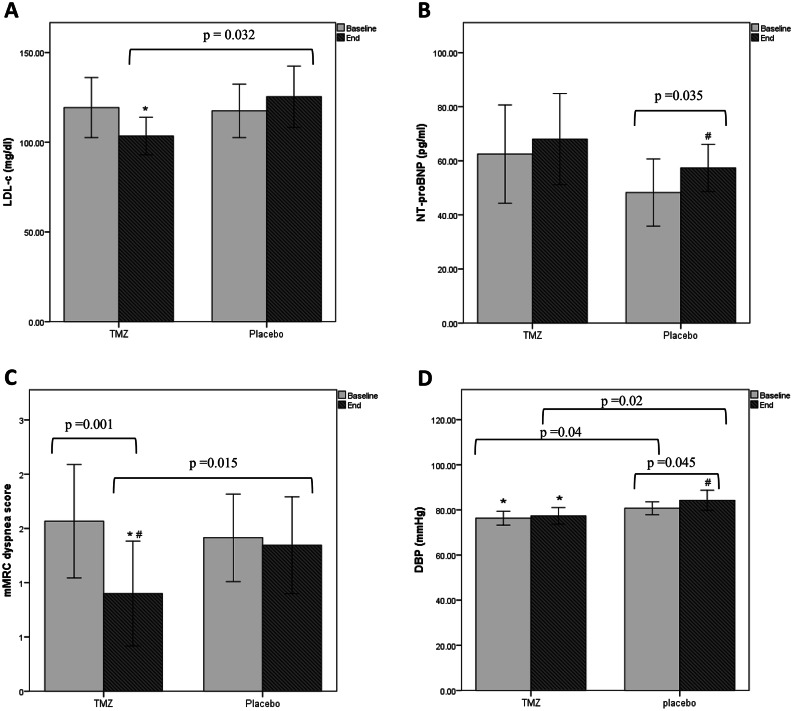
Change in laboratory and clinical parameters after 3 months of intervention in TMZ and placebo groups. Data are presented as mean ± 2 standard error. LDL-C: low density lipoprotein. NT-proBNP: n- terminal pro brain natriuretic peptide. mMRC: modified medical research council. DBP: diastolic blood pressure. * *p* < 0.05 vs. placebo group. ^#^*p* < 0.05 vs. baseline.

At baseline, all glycemic control and lipid profile parameters were comparable between the two study groups. After 3 months of intervention, the TMZ group exhibited lower LDL-C levels compared to the placebo group (*p* = 0.032). (Fig. 2) However, no statistically significant difference was found neither between groups nor within the same group regarding FBG, HbA1c%, total cholesterol, HDL-C, and TG. (Table 2)

**Table 2:** Comparison of biochemical and clinical parameters at baseline and after 3 months of treatment for the study groups.

Variables		TMZ group (*n* = 30)	Placebo group (*n* = 29)	*p*-value
FBG (mg/dl)	Baseline	146 (124.7–217.25)	156 (126.5–189)	0.921^a^
	After 3 months	150.5 (108- 203.5)	159 (112–195)	0.838^a^
	% change	−2.22 (−21.87 −12.42)	0 (−22.44–19.12)	0.638^a^
HbA1c %	Baseline	7.44 ± 1.87	8.15 ± 1.94	0.157^b^
	After 3 months	7.51 ± 1.59	7.8 ± 2.18	0.559^b^
	% change	0 (−7.74–9.89)	−4.28 (−21.13-10.06)	0.115^a^
TC (mg/dl)	Baseline	184.51 ± 47.37	182.33 ± 45.42	0.859^b^
	After 3 months	174.41 ± 30.51	193.35 ± 51.62	0.096^b^
	% change	−1.43 ± 21.87	2.14 ± 27.56	0.583^b^
LDL-C (mg/dl)	Baseline	119.29 ± 45.05	115.1 ± 40.67	0.711^b^
	After 3 months	103.43 ± 28.31	125.34 ± 45.27	0.032*^b^
	% change	−4.72 ± 34.49	7.69 ± 39.69	0.204^b^
HDL-C (mg/dl)	Baseline	40.62 ± 8.65	40.68 ± 10.69	0.979^b^
	After 3 months	43.89 ± 8.47	40.43 ± 8.58	0.979^b^
	% change	11.92 ± 31.41	−0.63 ± 33.72	0.144^b^
TG (mg/dl)	Baseline	115 (96–153)	126.9 (87.5–148.5)	0.975^a^
	After 3 months	128 (102.5- 153.5)	120 (95–169.75)	0.528^a^
	% change	1.29 (−16.13 −30.08)	7.14 (−15.69 −33.01)	0.868^a^
TNF-α (ng/L)	Baseline	112.5 (67.54–143.87)	95.88 (57.73 −130.88)	0.332^a^
	After 3 months	107.28 (72.52–168.61)	88.92 (63.08–100.31)	0.059^a^
	% change	2.64 (−13.79–26.61)	27.89 (−22.12 −61.49)	0.739^a^
TGF-β1(ng/ml)	Baseline	6.32 (4.65–12.75)	6.61 (4.63–8.66)	0.808^a^
	After 3 months	7.37 (5.19–12.24)	7.54 (5.66–9.22)	0.622^a^
	% change	11.09 (−11.54–62.32)	27.89 (−22.12–61.49)	0.988^a^
Nt-proBNP (pg/ml)	Baseline	47.3 (31.76–73.24)	39.4 (30.63–63.31)	0.231^a^
	After 3 months	60.31 (34.89–76.3)	57.28 (38.52–70.86)	0.802^a^
	% change	9.71 (−6.22–39.73)	17.22 (−6.85 −83.14)	0.310^a^
mMRC dyspnea score	Baseline	1 (0–3)	1 (1–3)	0.055^a^
	After 3 months	0 (0–1)	1 (0.5–3)	0.015^*a^
SBP (mm Hg)	Baseline	123.33 ± 12.67	130.38 ± 10.89	0.026^*b^
	After 3 months	124.87 ± 15.5	131.03 ± 14.89	0.125^b^
	% change	1.75 ± 12.38	0.68 ± 11.57	0.732^b^
DBP (mm Hg)	Baseline	76.33 ± 8.4	80.76 ± 7.72	0.04^*b^
	After 3 months	77.33 ± 10.12	84.24 ± 11.98	0.02^*b^
	% change	1.87 ± 13.16	4.31 ± 10.55	0.437^b^

Data were presented as mean ± standard deviation or median (interquartile range); FBG: fasting blood glucose; HbA1c: glycated hemoglobin; TC: total cholesterol; LDL-C: low-density lipoprotein; HDL-C: high-density lipoprotein; TG: triglycerides; TNF-α: tumor necrosis factor alpha; TGF-β1: transforming growth factor beta 1; NT-proBNP; N- terminal pro brain natriuretic peptide; mMRC: modified Medical Research Council; SBP: systolic blood pressure; DBP: diastolic blood pressure.

Statistical tests: ^a^ Mann-Whitney test, p-value ≥ 0.05; ^b^ Un-paired student t-test, p-value ≥ 0.05; * p-value < 0.05 is statistically significant.

A multiple linear regression model was conducted to evaluate the effect of TMZ compared to placebo on LDL-c after adjusting for confounders, TMZ group showed a statistically significant improvement in LDL-C compared to the placebo group (β= −18.657, *p* = 0.045). (Supplementary Table 1)

### Inflammatory and cardiac fibrosis markers and NT-proBNP

The levels of TNF-α and TGF-β1 were comparable at baseline in the two study groups and there were no significant changes in these parameters after the 3 months study period in both groups (Table 2).

At baseline, the levels of NT-proBNP were comparable between both groups, (Table 2). Compared to baseline, the two study groups exhibited higher levels of NT-proBNP after 3 months of treatment but the increase was statistically significant only in the placebo group (*p* = 0.035), (Fig. 2).

### Clinical parameters

**Fig. 3:**
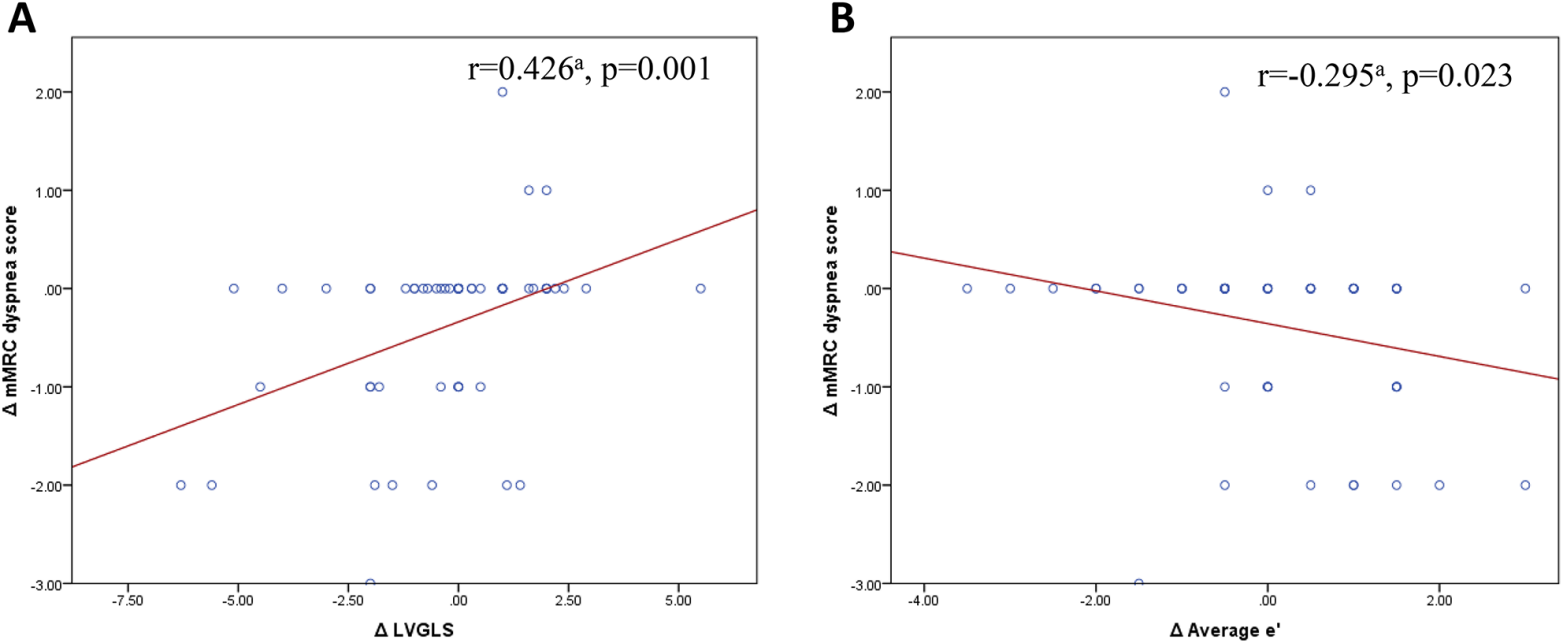
Bivariate correlation analysis of, (A) Δ mMRC dyspnea score versus Δ LVGLS and (B) Δ mMRC dyspnea score versus Δ average e’. mMRC: modified medical research council. LVGLS: left ventricular global longitudinal strain. e’: peak early diastolic velocity of mitral annular motion. ^a^ Spearman’s rho correlation coefficient.

At baseline, the two groups were comparable regarding mMRC dyspnea score, but the placebo group had significantly higher SBP and DBP (p = 0.026 and p = 0.04, respectively). After 3 months of intervention, the mMRC dyspnea score decreased significantly in the TMZ group while it remained unchanged in the placebo group. Though SBP didn’t change in both groups, DBP increased significantly in the placebo group while it remained unchanged in the TMZ group, (Fig. 2). However, this difference didn’t reach statistical significance when comparing the percent change in both groups, (Table 2). On bivariate correlation analysis, Δ mMRC dyspnea score showed significant positive correlation with ΔLVGLS (r = 0.425, p = 0.001) and significant negative correlation with Δ average e’ (*r*=−0.295, *p* = 0.023), (Fig. 3).

### Echocardiographic parameters

**Fig. 4:**
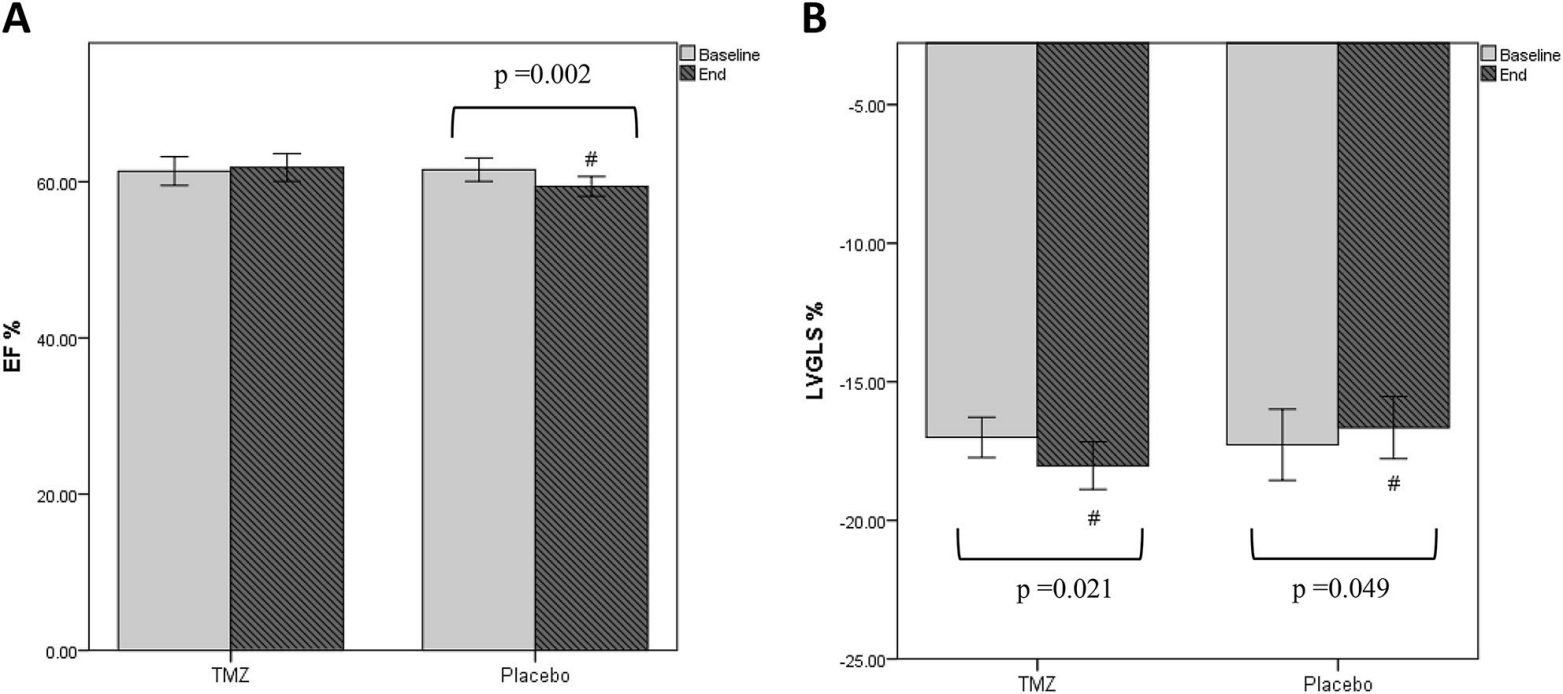
Change in systolic function parameters after 3 months of intervention in TMZ and placebo groups. Data are presented as mean ± 2 standard error. EF: ejection fraction. LVGLS: left ventricular global longitudinal stain. ^#^*p* < 0.05 vs. baseline.

**Fig. 5:**
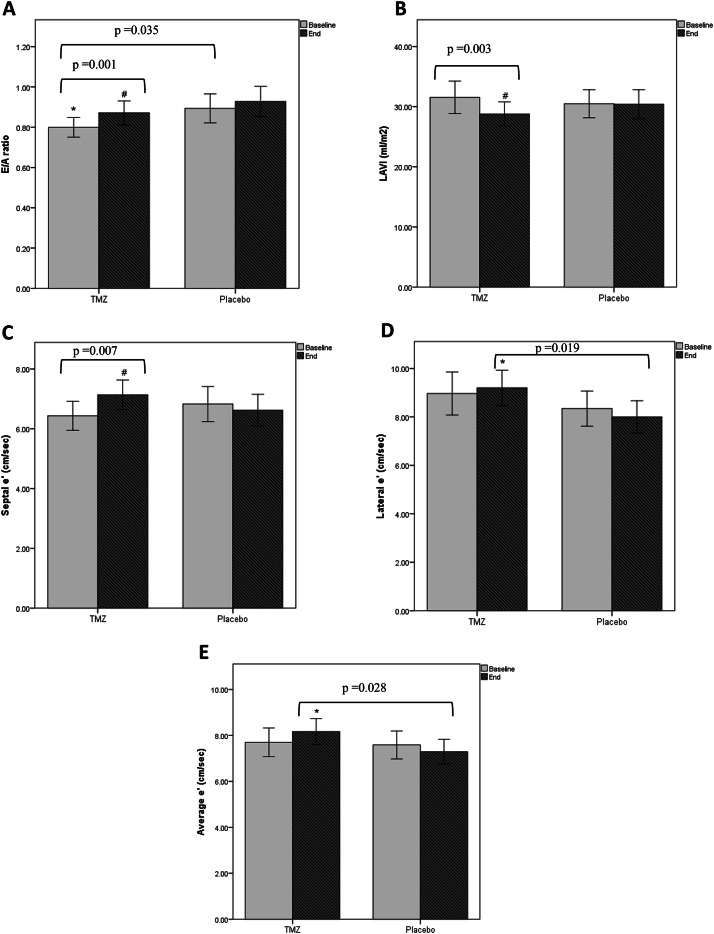
Change in diastolic function parameters after 3 months of intervention in TMZ and placebo groups. Data are presented as mean ± 2 standard error. E/A: peak early to late trans-mitral flow velocity. LAVI: left atrial volume index. e’: peak early diastolic velocity of mitral annular motion. ^#^*p* < 0.05 vs baseline. * *p* < 0.05 vs placebo group.

A comparison of echocardiographic parameters at baseline and after 3 months of treatment in both groups is shown in (Table 3). At baseline, both groups were comparable regarding LV dimensions, LAD, LVMI, RWT and remained statistically comparable after the 3-month intervention period. Regarding systolic function parameters, both groups were comparable at baseline, but the placebo group showed a significant decrease in EF% at the end of the study, (Fig. 4). However, this difference didn’t reach statistical significance when comparing the percent change in both groups. Regarding mitral inflow velocities, the TMZ group had a higher A-wave and lower E/A ratio at baseline. By the end of the study, a significant increase in the E/A ratio was noted in the TMZ group, (Fig. 5), but no significant difference was detected when comparing the percent change in both groups. Similarly, in multiple regression analysis, TMZ did not have a statistically significant effect on the E/A ratio (β = 0.019, p = 0.548) after adjusting for the other confounders. (Supplementary Table 2) Concerning diastolic function parameters, both groups were comparable in LAVI, septal e’, lateral e’, average e’ and E/e’ average ratio at baseline but after 3 months of intervention, a significant decrease in LAVI and a significant increase in septal e’ were noted in the TMZ group. Additionally, the TMZ group showed a statistically significant higher lateral e’ and average e’ compared to the placebo group, however the E/e’ average ratio remained unchanged, (Fig. 5). This improvement in the diastolic function of the TMZ group was further confirmed by finding a significant difference in the percent change of LAVI (*p* = 0.034, 95% CI=−14.72- −0.59), septal e’ (*p* = 0.007, 95% CI = 2.66–22.54) and average e’ (*p* = 0.015, 95% CI = 2.22–19.68).

**Table 3:** Comparison of echocardiographic parameters at baseline and after 3 months of treatment for the study groups.

**Variables**		**TMZ group (n = 30)**	**Placebo group (n = 29)**	**p-value**
LVEDD (mm)	Baseline	48.73 ± 3.81	49.24 ± 5.65	0.686^a^
	After 3 months	49.36 ± 3.56	49.52 ± 3.69	0.874^a^
	% change	1.54 ± 6.78	1.15 ± 7.29	0.83^a^
LVESD (mm)	Baseline	30.7 ± 3.16	30.89 ± 4.78	0.853^a^
	After 3 months	30.53 ± 3	31.34 ± 4.02	0.382^a^
	% change	−0.01 ± 10.14	2.06 ± 8.9	0.408^a^
LAD (mm)	Baseline	40.3 ± 5.07	39.48 ± 4.03	0.497^a^
	After 3 months	39 ± 4.99	40.07 ± 4.48	0.391^a^
	% change	−2.65 ± 11.32	1.91 ± 10.66	0.117^a^
RWT	Baseline	0.39 ± 0.04	0.401 ± 0.04	0.552^a^
	After 3 months	0.39 ± 0.05	0.41 ± 0.04	0.263^a^
	% change	−0.09 ± 10.32	2.86 ± 15.93	0.404^a^
LVMI (g/m^2^)	Baseline	85.94 ± 14.77	86.68 ± 18.96	0.868^a^
	After 3 months	88.52 ± 14.97	91.18 ± 20.18	0.567^a^
	% change	5.19 ± 22.99	6.44 ± 18.45	0.819^a^
LVEDVI (ml/m^2^)	Baseline	39.88 ± 7.49	41.79 ± 9.38	0.392^a^
	After 3 months	38.4 ± 6.8	38.51 ± 8.34	0.957^a^
	% change	−2.48 ± 14.59	−6.39 ± 14.42	0.304^a^
LVESVI (ml/m^2^)	Baseline	15.44 ± 3.47	16.71 ± 4.62	0.237^a^
	After 3 months	14.92 ± 3.64	15.83 ± 4.29	0.382^a^
	% change	−1.56 ± 22.14	−2.58 ± 20.87	0.856^a^
EF %	Baseline	61.36 ± 5.02	61.55 ± 4.06	0.877 ^a^
	After 3 months	61.83 ± 4.89	59.41 ± 3.41	0.032^*a^
	% change	1.59 ± 13.12	−3.28 ± 5.26	0.068^a^
E (cm/s)	Baseline	69.86 ± 14.67	70.24 ± 17.37	0.929 ^a^
	After 3 months	73.76 ± 16.25	72.62 ± 17.86	0.797^a^
	% change	6.75 ± 17.64	5.2 ± 21.59	0.763^a^
A (cm/s)	Baseline	87.66 ± 13.74	79 ± 12.45	0.014*^a^
	After 3 months	85.26 ± 14.27	78.83 ± 13.63	0.082^a^
	% change	−2.22 ± 10.84	0.04 ± 9.64	0.402^a^
E/A ratio	Baseline	0.79 ± 0.13	0.89 ± 0.19	0.035*^a^
	After 3 months	0.87 ± 0.16	0.93 ± 0.2	0.237^a^
	% change	9.15 ± 13.29	5.04 ± 18.47	0.329^a^
DT (ms)	Baseline	190.7 ± 35.82	193.41 ± 43.31	0.794^a^
	After 3 months	194.76 ± 34.37	198.03 ± 39.31	0.737^a^
	% change	4.37 ± 22.12	2.39 ± 32.91	0.786^a^
LAVI (ml/m^2^)	Baseline	31.54 ± 7.36	30.48 ± 6.28	0.555^a^
	After 3 months	28.77 ± 5.55	30.39 ± 6.53	0.307^a^
	% change	−6.99 ± 13.68	0.66 ± 13.39	0.034*^a^
Septal e’ (cm/s)	Baseline	6.43 ± 1.33	6.82 ± 1.58	0.304^a^
	After 3 months	7.13 ± 1.36	6.62 ± 1.42	0.162^a^
	% change	13.06 ± 19.92	−0.96 ± 18.35	0.007*^a^
Lateral e’ (cm/s)	Baseline	8.96 ± 2.44	8.34 ± 1.95	0.285^a^
	After 3 months	9.2 ± 2.01	8 ± 1.79	0.019*^a^
	% change	6.69 ± 23.45	−2.09 ± 19.63	0.124^a^
Average e’ (cm/s)	Baseline	7.7 ± 1.71	7.58 ± 1.63	0.795^a^
	After 3 months	8.16 ± 1.53	7.29 ± 1.45	0.028*^a^
	% change	8.46 ± 18.64	−2.49 ± 14.52	0.015*^a^
Average E/ e’ ratio	Baseline	9.51 ± 3.21	9.48 ± 2.37	0.969 ^a^
	After 3 months	9.23 ± 2.34	10.19 ± 2.9	0.167^a^
	% change	0.03 ± 17.96	9.39 ± 23.91	0.094^a^
LVGLS %	Baseline	−17.01 ± 1.98	−17.27 ± 3.45	0.717^a^
	After 3 months	−18.02 ± 2.35	−16.65 ± 3.02	0.056^a^
	% change	6.66 ± 13.88	−2.79 ± 9.64	0.004*^a^

Data were presented as mean ± standard deviation; LVEDD: left ventricular end diastolic dimension; LVESD: left ventricular end systolic dimension; LAD: left atrial diameter; LVMI: left ventricular mass index; RWT: relative wall thickness; LVEDVI: left ventricular end diastolic volume index; LVESVI: left ventricular end systolic volume index; EF: ejection fraction; E: peak early trans-mitral flow velocity ; A:peak late trans-mitral flow velocity; DT: deceleration time; LAVI: left atrial volume index; e’: peak early diastolic velocity of mitral annular motion; LVGLS: left ventricular global longitudinal strain. Statistical tests: a Un-paired student t-test, p-value ≥ 0.05; * p-value< 0.05 is statistically significant.

A multiple linear regression model was conducted to evaluate the effect of TMZ compared to placebo on diastolic function parameters. After adjusting for confounders, the TMZ group showed a statistically significant improvement in average e’ (β = 1.006, *p* > 0.001) (Supplementary Table 3) and LAVI (β=−2.156, *p* = 0.032) compared to the placebo group. (Supplementary Table 4)

### Left ventricular global longitudinal strain

At baseline, LVGLS was comparable in the study groups. Compared to baseline, the TMZ group exhibited a significant increase (*p* = 0.021) while the placebo group exhibited a significant decrease (*P* = 0.049) in the absolute value of LVGLS, (Fig. 4). Moreover, this result was further confirmed by analyzing the difference in the percent change from baseline between groups (*p* = 0.004, 95% CI = 3.21–15.71), (Table 3). To account for baseline confounders, a multiple linear regression model was employed, and the TMZ group still demonstrated a statistically significant improvement in LVGLS (β=−1.715, *p* = 0.001). (Supplementary Table 5)

### Safety assessment of trimetazidine

No serious side effects were reported throughout the study period. The most reported side effects were mild gastrointestinal side effects that were comparable between the two study groups.

## Discussion

The current study demonstrated that the use of TMZ in type 2 diabetic patients with LV diastolic dysfunction for 3 months resulted in a significant improvement in LDL-C levels, mMRC dyspnea score, and LV diastolic function parameters as assessed by E/A ratio, e’ velocity and LAVI in parallel with a positive impact on LV systolic function parameters as assessed by EF and LVGLS. However, TMZ use did not show any significant changes in the levels of TNF-α, TGF-β1 and NT-proBNP. To the best of our knowledge, this is the first study to report the effects of TMZ treatment on the early phases of DCM.

### Metabolic effects of trimetazidine

Diabetic dyslipidemia is considered to be the major cause of the increased cardiovascular risk in diabetic patients^25^. In the current study, TMZ showed a positive effect on LDL-C levels which is consistent with the findings of a prior study in type 2 diabetic patients with ischemic cardiomyopathy^26^. These favorable metabolic effects of TMZ could be explained by the findings of Zhang et al. who demonstrated that TMZ treatment in a non‑alcoholic fatty liver disease mouse model alleviated hyperlipidemia, glucose intolerance and hepatic steatosis via the upregulation of AMP‑activated protein kinase (AMPK)- forkhead box O1 (FOXO1) pathway^27^.

### Effect of trimetazidine on cardiac biomarkers

Trimetazidine has a well-documented anti-inflammatory effect in preclinical studies^15,28^. Su et al. showed that TMZ exerts its cardioprotective effect via inhibition of Programmed Cell Death factor 4 (PDCD4)/ Nuclear Factor Kappa B (NF-κB) / TNF-α pathway in cardiomyocytes^15^. Furthermore, TMZ treatment resulted in a significant decrease in serum TNF- α levels in ischemic cardiomyopathy patients^29^. In contrast, our results did not show an effect of TMZ on TNF-α levels. The discrepancy between the two studies could be attributed to the fact that Su et al. recruited ischemic cardiomyopathy patients with EF < 40% whose mean baseline TNF-α levels were higher compared to our study that recruited patients with early DCM and higher EF. Therefore, subjects of the current study may have been simply too early in the disease process to demonstrate a detectable alteration in TNF-α levels with a similar TMZ therapeutic regimen.

Cardiac fibrosis has been identified as the key regulator of cardiac remodeling. TGF-β acts as a “master of all trades” when it comes to cardiac fibrosis, where it can induce cardiac hypertrophy and cardiomyocyte death^30^. TGF-β proved effectiveness in the early diagnosis of DCM, where diabetic patients with LV diastolic dysfunction exhibited higher plasma levels of TGF-β in comparison to diabetic patients without LV diastolic dysfunction^31^. TMZ has been shown to alleviate myocardial fibrosis in several preclinical studies by modulating the mitogen-activated protein kinase (MAPK) pathway^16^, downregulating the protein expression of Collagen Ι, Collagen III, Connective tissue growth factor (CTGF)^17^, reducing gene expression of CTGF, TGF-β1, smad2 and smad3^32^. However, the effect of TMZ on cardiac fibrosis is less documented in clinical studies, only one recent clinical study in ischemic cardiomyopathy patients showed that TMZ treatment caused a significant reduction in CTGF levels^29^. On the contrary, the current study showed no effect of TMZ on TGF-β1 levels, however, this neutral effect of TMZ on serum TGF-β1 does not imply a similar effect on myocardial TGF-β1 owing to the previous findings of Lok et al. showing that the increase in myocardial TGF-β1 mRNA expression is not reflected on plasma TGF-β1 levels^33^.

Natriuretic peptides (BNP and NT-proBNP) are generally secreted in response to the mechanical stretch of myocardial walls induced by volume expansion or pressure overload^34^. NT-proBNP is the inactive terminal molecule of BNP, it has a longer half-life (90 to 120 min) and is thought to have better plasma stability making it the preferable laboratory marker^35^. The role of natriuretic peptide in screening of preclinical DCM has been controversial^36–38^, . Yet, higher BNP levels even within the normal range were associated with a poor prognosis^38^. In the current study, diabetic patients with diastolic dysfunction exhibited normal NT-proBNP levels *<*125 pg/mL as per the recommendation of the Heart Failure Association of the European Society of Cardiology^34^. The current study showed that TMZ had a neutral effect on NT-proBNP which is in line with the findings of recent reports in patients with non-ischemic heart failure^39^and heart failure with preserved ejection fraction (HFpEF)^40^. However, in the present study, NT-proBNP levels increased significantly in the placebo group. Since natriuretic peptides levels increase in response to the increased volume and pressure overload on the myocardial wall, this increase is assumed to occur in response to the observed increase in DBP which is in line with the findings of Kir et al. who revealed that BNP levels are higher in type 1 diabetic children with elevated DBP load compared with those with normal DBP load^41^.

### Effect of trimetazidine on dyspnea severity

In the current study, more than two thirds of the patients experienced dyspnea with daily living activities, although previous reports showed that patients in early phases of DCM were usually asymptomatic^42^. However, obesity has been shown to be highly associated with dyspnea^43^and since a great proportion of our patients were obese, obesity may be the primary cause of dyspnea. Yet, Santos et al. found that self-reported dyspnea in patients free from cardiopulmonary diseases has been independently associated with greater risk of incident heart failure^44^. The mMRC dyspnea scale has proved effectiveness in assessment of dyspnea severity of daily living in obese patients^45^, heart failure patients^46^and patients with chronic obstructive pulmonary diseases^47^. Moreover, Launois et al. demonstrated an association between exercise tolerance assessed by 6-minute walking distance (6MWD) and the mMRC dyspnea score^45^. The current study showed that TMZ administration significantly improved the mMRC dyspnea score, which is in line with the findings of a recent meta-analysis that showed the positive effects of TMZ on New York Heart Association (NYHA) functional class and 6MWD in patients with heart failure and reduced ejection fraction (HFrEF)^48^. Additionally, the degree of dyspnea was associated with increased LV filling pressure and impaired LVGLS in patients with type 1 DM and normal EF^49^. Similarly, the present study demonstrated that the change in dyspnea severity was correlated with the change in LVGLS and e’, indicating that these preliminary promising effects of TMZ on the severity of dyspnea are reflective and could be attributed to its positive effect on LV systolic and diastolic functions.

#### Effect of trimetazidine on diastolic function and LVGLS

Left ventricular diastolic dysfunction is the hallmark of early DCM that occurs due to increased FA oxidation in diabetic hearts. FAs stimulate the uncoupling proteins in the mitochondria and raise the oxygen demand of the mitochondria for the synthesis of ATP, that in turn compromises myocyte contraction and diastolic function because of the impairment in myocardial energy production^50^. TMZ works as a metabolic switch, inducing cardiac myocytes to use glucose as an energy substrate over free FAs^13^, therefore, it is assumed that TMZ could exert a beneficial effect on LV diastolic dysfunction seen in DCM. In the current study, TMZ enhanced LV diastolic dysfunction measures which is consistent with the findings of previous studies in diabetic patients with ischemic cardiomyopathy^24^and HFrEF patients^51^. Though LV diastolic dysfunction is traditionally assumed to be the first myocardial change in DCM, recent research has demonstrated that LV systolic dysfunction can exist in the early stages of DCM. STE is a relatively new technique that measures myocardial strain, or the deformation of particular myocardial regions during a cardiac cycle, to evaluate subclinical cardiac function^52^. In previous cohort studies of diabetic patients, about half of the patients had evidence of subclinical left ventricular dysfunction using GLS, despite having normal EF. Importantly, a lower GLS detected by STE was correlated with cardiovascular events and provided additional prognostic value up to ten years after revealing^53,54^. Therefore, STE can improve the early diagnosis of subclinical DCM^11^. The positive effect of TMZ on LV systolic function as assessed by EF has been proved in many previous studies in non-ischemic^39^and ischemic cardiomyopathy^29,51^. Moreover, Harjoko et al. observed that the favorable effects of TMZ on LV systolic function are also evident on GLS^51^which is consistent with the findings of the current study that reported a significant improvement in LVGLS in the TMZ treated group. On the other hand, The DoPING-HFpEF trial showed that TMZ had neutral effects on both LV systolic and diastolic function parameters^40^. Given that HFpEF is a combination of phenotypes resulting from different pathophysiological mechanisms rather than a disease with a single etiology^55^ along with the difference in study design and the disparity between patients’ clinical characteristics in our study and the DoPING-HFpEF trial where the majority of patients in the DoPING-HFpEF were hypertensive without diabetes mellitus, this may account for the lack of beneficial effects of TMZ in the DoPING-HFpEF trial.

## Conclusion

The addition of TMZ to standard treatment of type 2 diabetic patients with LV diastolic dysfunction for three months was well-tolerated and effective in improving LV systolic and diastolic functions along with reducing dyspnea severity and LDL-C levels. However, TMZ had no effect on cardiac inflammatory or fibrosis markers nor NT-proBNP. Thus, in diabetic patients with evidence of LV diastolic dysfunction, early use of TMZ treatment may help slow the progression of DCM.

### Limitations

The current study has a modest number of subjects. Further large-scale studies are needed to expand our findings. Moreover, the duration of our study could be prolonged to explore the long-term effects of TMZ on biomarkers of inflammation and fibrosis as well as LV function. Furthermore, due to financial limitations, we were unable to measure the levels of additional biomarkers associated with DCM, such as galectin-3 and fibroblast growth factor 23, which could have provided further insights into the pathophysiological changes induced by TMZ therapy.

## Electronic Supplementary Material

Below is the link to the electronic supplementary material.

Supplementary Material 1

## Data Availability

The datasets generated during and/or analysed during the current study are available from the corresponding author on reasonable request.
