# Supplementary material for: Effect of trimetazidine on left ventricular functions and cardiac biomarkers in diabetic patients with left ventricular diastolic dysfunction: a randomized controlled trial
Source: Sci Rep. 2025 Jan 16;15:2115. doi: 10.1038/s41598-024-83213-w (PMC11736023; doi:10.1038/s41598-024-83213-w)
Supplement: Supplementary file 1 — Supplementary Material 1 [file 41598_2024_83213_MOESM1_ESM.docx]

**Supplementary Table 1: Multiple linear regression model fitted to describe the effect of trimetazidine on patients’ LDL-C levels by the end of the study after adjusting for baseline LDL-C level, patients’age, baseline HbA1c , statin and metformin usage.**

| Variable | Coefficient (β) | *p-value* | 95% confidence interval | Overall model test *p-value* |
| --- | --- | --- | --- | --- |
| TMZ group | -18.657 | 0.045^a*^ | -36.92- -0.394 | <0.001^b*^ |
| LDL-C at baseline | 0.351 | 0.001^a*^ | 0.144- 0.559 |  |
| Age | -0.112 | 0.863 ^a^ | -1.399- 1.176 |  |
| HbA1c at baseline | 6.549 | 0.006 ^a*^ | 1.921- 11.178 |  |
| Statin | -3.735 | 0.699 ^a^ | -23.013-15.543 |  |
| Metformin | -6.297 | 0.54 ^a^ | -26.809- 14.215 |  |
| TMZ: trimetazidine; LDL-C: low density lipoprotein; HbA1c: glycated hemoglobin; Statistical tests: ^a^ t- test, *p-value ≥* 0.05: non-significant; ^a*^ t- test, *p-value* < 0.05: significant; ^b*^ F- test, *p-value* < 0.05: significant | | | | |

**Supplementary Table 2: Multiple linear regression model fitted to describe the effect of trimetazidine on patients’ E/A ratio by the end of the study after adjusting for baseline E/A ratio, patients’age, baseline LDL-C level and baseline LVGLS.**

| Variable | Coefficient  (β) | *p-value* | 95% confidence interval | Overall model test *p-value* |
| --- | --- | --- | --- | --- |
| TMZ group | 0.019 | 0.548^a^ | -0.051-0.089 | <0.001^b*^ |
| E/A ratio at baseline | 0.771 | <0.001^a*^ | 0.555-0.988 |  |
| Age | -0.002 | 0.321 ^a^ | -0.008- 0.002 |  |
| LDL-C at baseline | 3.66×10^-4^ | 0.359 ^a^ | -4.28×10^-4^- 0.001 |  |
| LVGLS at baseline | -0.007 | 0.218 ^a^ | -0.021-0.004 |  |
| TMZ: trimetazidine; E: peak early trans-mitral flow velocity; A: peak late trans-mitral flow velocity; LDL-C: low density lipoprotein; LVGLS: left ventricular global longitudinal strain.  Statistical tests: ^a^ t- test, *p-value ≥*  0.05: non-significant; ^a*^ t- test, *p-value* < 0.05: significant; ^b*^ F- test, *p-value* < 0.05: significant | | | | |

**Supplementary Table 3 : Multiple linear regression model fitted to describe the effect of trimetazidine on patients’ average e’ by the end of the study after adjusting for baseline average e’ , patients’age, baseline HbA1c and baseline LVGLS.**

| Variable | Coefficient  (β) | *p-value* | 95% confidence interval | Overall model test *p-value* |
| --- | --- | --- | --- | --- |
| TMZ group | 1.006 | <0.001^a*^ | 0.473- 1.541 | <0.001^b*^ |
| Average e’ at baseline | 0.422 | <0.001^a*^ | 0.232- 0.611 |  |
| Age | -0.079 | <0.001^a*^ | -0.124- -0.034 |  |
| HbA1c at baseline | -0.03 | 0.679 ^a^ | -0.174- 0.114 |  |
| LVGLS at baseline | -0.132 | 0.016 ^a*^ | -0.239- -0.026 |  |
| TMZ: trimetazidine; e’: peak early diastolic velocity of mitral annular motion; HbA1c: glycated hemoglobin; LVGLS: left ventricular global longitudinal strain.  Statistical tests: ^a^ t- test, *p-value ≥*  0.05: non-significant; ^a*^ t- test, *p-value* < 0.05: significant; ^b*^ F- test, *p-value* < 0.05: significant | | | | |

**Supplementary Table 4 : Multiple linear regression model fitted to describe the effect of trimetazidine on patients’ LAVI by the end of the study after adjusting for baseline LAVI, patients’age, baseline LDL-C and baseline LVGLS.**

| Variable | Coefficient  (β) | *p-value* | 95% confidence interval | Overall model test *p-value* |
| --- | --- | --- | --- | --- |
| TMZ group | -2.156 | 0.032 ^a*^ | -4.123- -0.189 | <0.001^b*^ |
| LAVI at baseline | 0.695 | <0.001^a*^ | 0.541- 0.85 |  |
| Age | -0.014 | 0.847 ^a^ | -0.163- 0.134 |  |
| LDL-C at baseline | -0.015 | 0.188 ^a^ | -0.038- 0.007 |  |
| LVGLS at baseline | 0.282 | 0.149 ^a^ | 0.104- 0.669 |  |
| TMZ: trimetazidine; LAVI: left atrial volume index; LDL-C: low density lipoprotein; LVGLS: left ventricular global longitudinal strain.  Statistical tests: ^a^ t- test, *p-value ≥*  0.05: non-significant; ^a*^ t- test, *p-value* < 0.05: significant; ^b*^ F- test, *p-value* < 0.05: significant | | | | |

**Supplementary Table 5: Multiple linear regression model fitted to describe the effect of trimetazidine on patients’ LVGLS by the end of the study after adjusting for baseline LVGLS, patients’age, baseline LDL-C level and baseline E/A ratio.**

| Variable | Coefficient  (β) | *p-value* | 95% confidence interval | Overall model test *p-value* |
| --- | --- | --- | --- | --- |
| TMZ group | -1.715 | 0.001^a*^ | -2.732- -0.699 | <0.001^b*^ |
| LVGLS at baseline | 0.733 | <0.001^a*^ | 0.547-0.918 |  |
| Age | 0.048 | 0.221 ^a^ | 0.029- 0.1259 |  |
| LDL-C at baseline | 0.003 | 0.525 ^a^ | -0.007- 0.015 |  |
| E/A ratio at baseline | -0.142 | 0.928 ^a^ | -3.281- 2.997 |  |
| TMZ: trimetazidine; LVGLS: left ventricular global longitudinal strain; LDL-C: low density lipoprotein; E: peak early trans-mitral flow velocity; A: peak late trans-mitral flow velocity.  Statistical tests: ^a^ t- test, *p-value ≥*  0.05: non-significant; ^a*^ t- test, *p-value* < 0.05: significant; ^b*^ F- test, *p-value* < 0.05: significant | | | | |
